# Prestin-Driven Cochlear Amplification Is Not Limited by the Outer Hair Cell Membrane Time Constant

**DOI:** 10.1016/j.neuron.2011.04.024

**Published:** 2011-06-23

**Authors:** Stuart L. Johnson, Maryline Beurg, Walter Marcotti, Robert Fettiplace

**Affiliations:** 1 Department of Biomedical Science, University of Sheffield, Sheffield, S10 2TN, UK; 2 INSERM U587, Université Bordeaux Segalen, CHU Pellegrin, 33076 Bordeaux, France; 3 Department of Neuroscience, University of Wisconsin Medical School, Madison, WI 53706, USA

## Abstract

Outer hair cells (OHCs) provide amplification in the mammalian cochlea using somatic force generation underpinned by voltage-dependent conformational changes of the motor protein prestin. However, prestin must be gated by changes in membrane potential on a cycle-by-cycle basis and the periodic component of the receptor potential may be greatly attenuated by low-pass filtering due to the OHC time constant (τ_m_), questioning the functional relevance of this mechanism. Here, we measured τ_m_ from OHCs with a range of characteristic frequencies (CF) and found that, at physiological endolymphatic calcium concentrations, approximately half of the mechanotransducer (MT) channels are opened at rest, depolarizing the membrane potential to near −40 mV. The depolarized resting potential activates a voltage-dependent K^+^ conductance, thus minimizing τ_m_ and expanding the membrane filter so there is little receptor potential attenuation at the cell's CF. These data suggest that minimal τ_m_ filtering in vivo ensures optimal activation of prestin.

## Highlights

► Cochlear amplification via prestin limited by outer hair cell (OHC) time constant ► Endolymph Ca^2+^ opens half the transduction channels, depolarizing OHC to −40 mV ► Depolarization activates K^+^ conductance, reduces time constant and expands filter ► Minimal membrane filtering in vivo ensures prestin activation over all frequencies

## Introduction

Outer hair cells of the mammalian cochlea possess both sensory and motor functions, converting sound-induced vibrations of the basilar membrane into receptor potentials but also generating a mechanical output that augments motion of the basilar membrane and sharpens its frequency selectivity (Dallos, 1992; Fettiplace and Hackney, 2006). The motor capacity is often referred to as the cochlear amplifier for which two mechanisms have been proposed: somatic contractions and hair bundle motion. The rapid somatic contraction is attributable to the membrane protein prestin (Zheng et al., 2000; Dallos et al., 2008) that changes conformation according to membrane potential. Active motion of the hair bundle results from opening and adaptation of the mechanotransducer (MT) channels. This second mechanism is prominent in frogs and turtles (Martin and Hudspeth, 1999; Ricci et al., 2000) but signs of it have also been seen in mammals (Chan and Hudspeth, 2005; Kennedy et al., 2005). Several prestin mutants have been generated that reduce or abolish cochlear amplification (Liberman et al., 2002; Dallos et al., 2008) arguing that prestin has an obligatory role in the process.

A difficulty with the prestin hypothesis is that for it to implement feedback, it must be gated by changes in membrane potential on a cycle-by-cycle basis. However, the periodic component of the receptor potential may be greatly attenuated by low-pass filtering due to the OHC time constant, which has been reported to be at most a few hundred hertz (Housley and Ashmore, 1992; Preyer et al., 1994, 1996; Mammano and Ashmore, 1996). This problem does not exist in the hair bundle motor for which the speed is limited only by the feedback loop involving the MT channels, which includes the kinetics of their activation and fast adaptation. Several ways of circumventing the membrane time constant limitation of the somatic contraction mechanism have been advanced (reviewed in Ashmore, 2008) including gating of prestin by extracellular potentials (Dallos and Evans, 1995), by chloride influx evoked by stretch activation of the lateral membrane (Rybalchenko and Santos-Sacchi, 2003), or by considering current flow along the organ of Corti in a three-dimensional model (Mistrík et al., 2009). None of these has yet been validated experimentally.

Because OHCs possess a large voltage-dependent K^+^ conductance (Housley and Ashmore, 1992; Mammano and Ashmore, 1996), their time constant will depend on membrane potential and become smaller with activation of this conductance at depolarized potentials. Thus a crucial factor in determining the time constant for small perturbations is the OHC resting potential. The resting potential results largely from a balance between the two main ionic currents: an inward MT current and an outward voltage-dependent K^+^ current. MT currents in auditory hair cells display Ca^2+^-driven adaptation that dictates the fraction of the MT channels open at rest resulting in a sustained depolarizing current, which is larger when the hair bundles are exposed to low endolymphatic Ca^2+^ (Ricci et al., 1998). Previous estimates of the resting potential in OHCs have placed it at −60 to −70 mV (Mammano and Ashmore, 1996; Preyer et al., 1994; Marcotti and Kros, 1999). OHC resting potentials have also been measured in intact animals and again the most common value is ∼−70 mV (Dallos, 1985a; Russell et al., 1986).

Here, we report large ambient MT currents and receptor potentials in OHCs from acutely isolated cochleas. We measured τ_m_ from OHCs with different cochlear locations having CFs of 0.35–10 kHz. When hair bundles were exposed to endolymphatic Ca^2+^ (0.02 mM), about half of the mechanotransducer (MT) channels opened at rest, causing OHCs to depolarize to near −30 mV and, by activating a K^+^ conductance, lowered τ_m_. After adjustment for conditions existing in vivo, including endolymphatic potential and temperature, we estimate resting potentials of −40 mV and time constants at least ten times smaller than those previously reported. We propose that the OHC membrane time constant has been significantly overestimated and therefore no real limitation on the function of prestin may exist in vivo.

## Results

### The Effects of Endolymph Ca^2+^ on MT Currents

**Figure 1:**
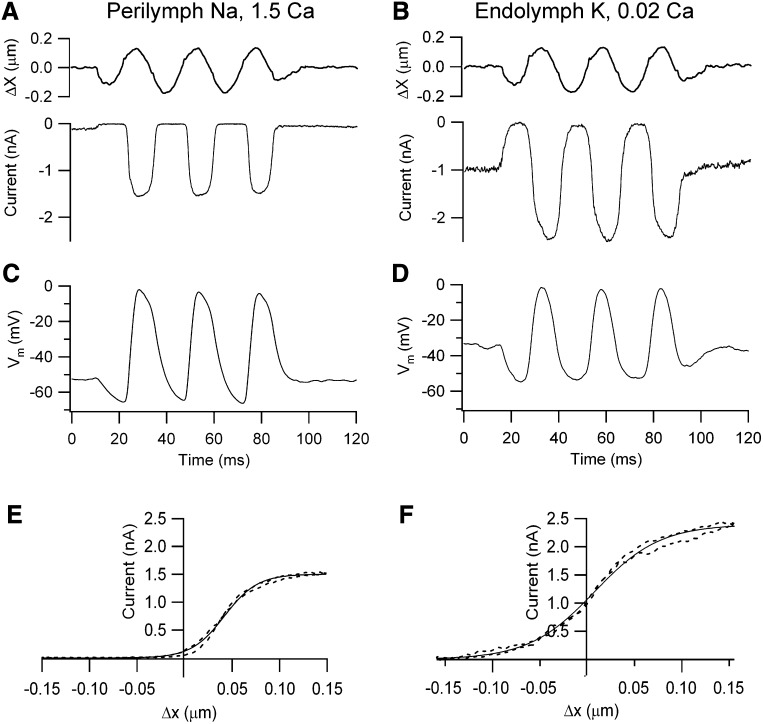
MT Currents and Receptor Potentials in Rat OHCs (A) Saturating receptor currents with hair bundles exposed to saline containing Na^+^ and 1.5 mM Ca^2+^. (B) Saturating receptor currents in a different OHC with hair bundles locally perfused with artificial endolymph containing K^+^ and 0.02 mM Ca^2+^. Note larger MT current amplitude and increased fraction on at rest compared to 1.5 mM Ca^2+^. (C) Receptor potentials, V_m_, for cell in (A) with hair bundles exposed to Na^+^, 1.5 mM Ca^2+^ saline. (D) Receptor potentials for cell in (B) with hair bundles exposed K^+^, 0.02 mM Ca^2+^ endolymph. The sinusoidal stimulus (top), which applies to both receptor currents and potentials, was delivered with a fluid jet, hair bundle motion being calibrated by projection on a photodiode pair; holding potential in voltage clamp −84 mV. (E) Relation between MT current (I) and bundle displacement (X) in (A) over one cycle of the response (dashed line). (F) MT current versus bundle displacement in (B) over one cycle of the response (dashed line). I–X results fitted with single Boltzmann (continuous line): I = I_MAX_/(1 + (exp((X_1_ − X)/X_e_)) where I_MAX_ = 1.51 nA, X_e_ = 0.017 μm, X_1_ = 0.04 μm (C, perilymph); I_MAX_ = 2.4 nA, X_e_ = 0.035 μm, X_1_ = 0.008 μm (D, endolymph). Apical cochlear location, P8 (A and C), P9 (B and D) rats, T = 22°C.

Local application to the OHC hair bundles of an endolymph-like solution, containing K^+^ as the monovalent cation and low, 0.02 mM Ca^2+^, had two effects on MT currents: it increased both the maximum current amplitude and the fraction of that current activated at rest (Figure 1). External Ca^2+^ is known to block the MT channels with a half-blocking concentration of 1 mM (Ricci and Fettiplace, 1998; Beurg et al., 2010) and the increased current amplitude in low Ca^2+^ stems from relief of this block. Moreover, the increased resting current in the presence of endolymphatic Ca^2+^ agrees with previous findings showing that when extracellular Ca^2+^ was increased it caused adaptation, thus closing some MT channels, whereas when Ca^2+^ influx was decreased, either by lowering its extracellular concentration or depolarizing to near the Ca^2+^ equilibrium potential, the open probability of the MT channels increased (Assad et al., 1989; Crawford et al., 1991; Ricci et al., 1998; Beurg et al., 2010). The initial characterization of the MT current was performed from OHCs located in the apical region of the rat cochlea (CF = 4 kHz) at room temperature (Figures 1A and 1B). In these OHCs, the maximum MT current increased from 1.2 ± 0.1 nA (n = 5) in normal perilymph-like saline to 2.1 ± 0.1 nA (n = 7) in endolymph and the fraction of current at rest increased from 0.06 ± 0.01 to 0.46 ± 0.01 (n = 7). The increase in the MT channel resting open probability caused by the endolymph-like solution is attributable solely to the reduced Ca^2+^ concentration and not to the use of endolymphatic high K^+^, which could itself cause OHC depolarization if allowed to reach the cell's basolateral membrane. If the perfusate contained 0.02 Ca^2+^ and Na^+^ instead of K^+^, the fraction of current at rest in rat apical OHCs (0.46 ± 0.08, n = 6) was similar to that obtained with K^+^. This indicates that the perfusion of the endolymph-like K^+^ solution was restricted to the hair bundles. In either case the consequence was a large standing MT current in low Ca^2+^ and in the absence of stimulation. The presence of this standing inward current in the endolymph solution (0.94 ± 0.04 nA), which may be termed a “silent current” by analogy with the dark current in photoreceptors (Baylor et al., 1979), meant that OHCs exhibited a significantly more depolarized membrane potential (−34 ± 3 mV, n = 7: p < 0.002) compared to that in perilymph (−51 ± 2 mV, n = 5). Because the MT current in OHCs has a reversal potential near 0 mV (Kros et al., 1992; Beurg et al., 2006), only a small electrical driving force exists in isolated preparations that lack the 90 mV endolymphatic potential. Nevertheless, substantial receptor potentials of 40–60 mV could be measured under current clamp conditions (Figures 1C and 1D). The mean receptor potential for a saturating stimulus was 51 ± 8 mV (n = 5) in perilymph and 42 ± 2 mV (n = 7) in endolymph. These responses were obtained for 0.1–0.2 μm maximum hair bundle displacements giving current-displacement relations that could be fit by a single Boltzmann (Figures 1E and 1F).

**Figure 2:**
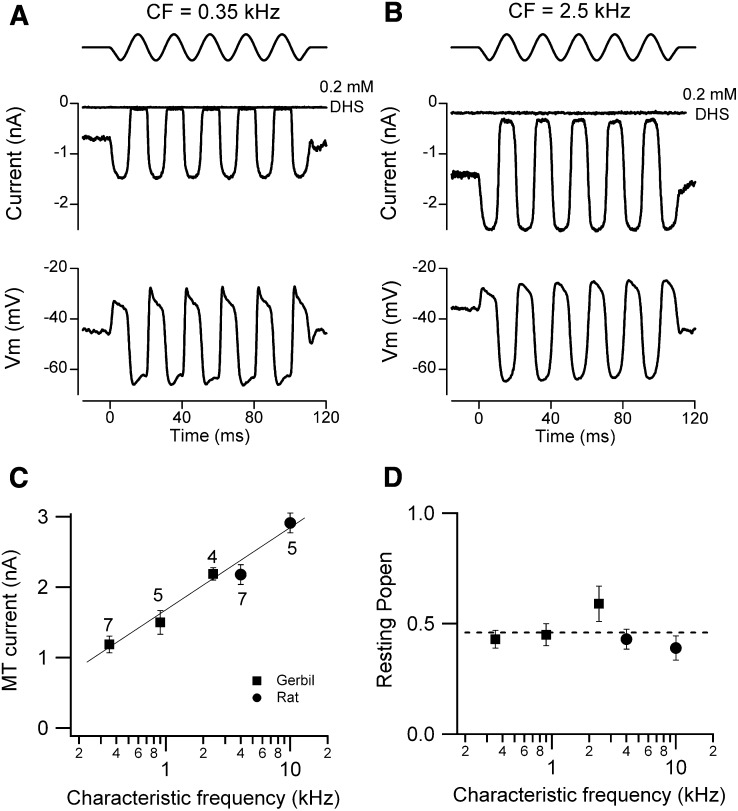
Tonotopic Variation in MT Currents with Hair Bundles Exposed to Endolymph (0.02 mM) Ca^2+^ (A) Saturating MT currents and receptor potentials in an apical gerbil OHC (CF = 0.35 kHz). (B) Saturating MT currents and receptor potentials in a gerbil middle turn OHC (CF = 2.5 kHz). In both (A) and (B), about half the maximum MT current was activated at rest and the current was abolished by addition of 0.2 mM DHS. Top traces show driving voltage to piezo. (C) MT current (mean ± SEM) versus CF for three gerbil locations (filled squares) and two rat locations (filled circles), number of measurements indicated by each point. (D) Resting open probability (*P*_open_; mean ± SEM) of the MT channels for gerbil and rat locations (P7–P10 animals). Currents recorded at −84 mV holding potential, T = 22°C–24°C.

Similar effects on the transduction current and receptor potential were also seen in OHCs of the isolated gerbil cochlea on exposing the hair bundles to endolymphatic Ca^2+^. MT currents measured at room temperature in the gerbil apex (CF = 0.35 kHz) increased from 0.67 ± 0.01 nA in normal 1.3 mM Ca^2+^ (n = 5) to 1.19 ± 0.05 nA in endolymph 0.02 mM Ca^2+^ (n = 7) and the fraction of current activated at rest increased under the same circumstance from 0.08 ± 0.01 to 0.43 ± 0.04. The increase in standing current in low Ca^2+^ was not attributable to activation of other conductances because it was fully abolished (Figures 2A and 2B) by addition of 0.2 mM dihydrostreptomycin (DHS), a known blocker of the OHC MT channel.

### Tonotopic Gradient in MT Conductance

In both gerbils and rats, the size of the MT current increased systematically with the CF of the OHC. Measurements were made at multiple cochlear locations, the CFs of which were interpolated from existing frequency maps for the two rodents (Müller, 1991, 1996). The two animal species were chosen because they have different but overlapping frequency ranges, the gerbil from 0.2–35 kHz and the rat from 1–55 kHz. Examples of MT currents in low Ca^2+^ for the gerbil are shown in Figures 2A and 2B. In the presence of 0.02 Ca^2+^ and either Na^+^ or K^+^, apical-coil gerbil OHCs (0.9 kHz) exhibited a similar MT current (Na^+^: 1.56 ± 0.25 nA, n = 5; K^+^: 1.52 ± 0.16 nA, n = 5) and MT channel resting open probability (Na^+^: 0.46 ± 0.01, n = 5; K^+^: 0.45 ± 0.05 nA, n = 5), confirming that the effects seen in the presence of the endolymph-like solution are only due to the low Ca^2+^ concentration. Collected results (Figure 2C) demonstrate a continuous increase in the MT current across the two animal species that is dependent only on the CF. Thus the MT current in the middle region of the gerbil cochlea (CF = 2.5 kHz) is very similar to that near the apex of the rat cochlea (CF = 4 kHz), both being measured at the same holding potential (−84 mV). Overall there was about a 3-fold increase in MT current as the CF increased from 0.35 to 10 kHz. An increase in the size of the MT current along the tonotopic axis has also been reported in the gerbil hemi cochlea (He et al., 2004). Despite the change in current amplitude, the fraction activated at rest (the resting Popen) in low Ca^2+^ was invariant with CF and had a mean of 0.46 ± 0.03 (Figure 2D). As a consequence the silent current increased in parallel with the maximum MT current.

**Figure 3:**
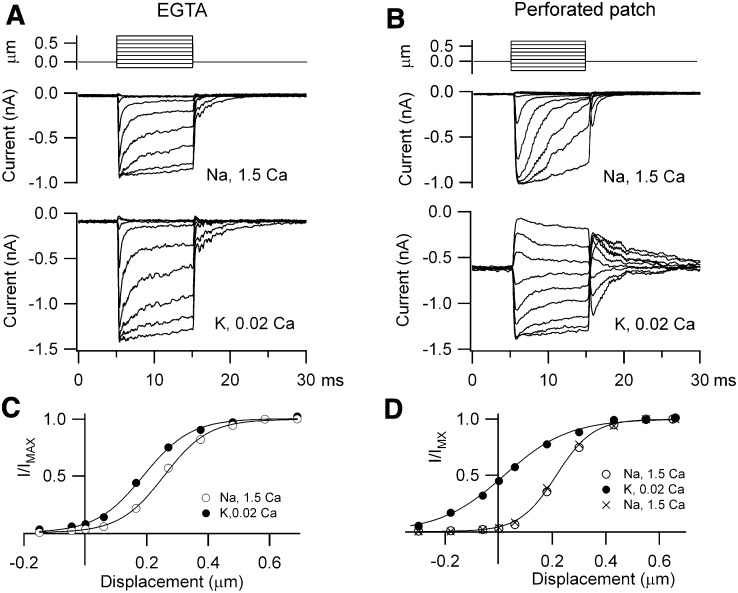
OHC Endogenous Ca^2+^ Buffer Assayed with Perforated Patch (A) MT currents recorded with an intracellular solution containing 1 mM EGTA as the Ca^2+^ buffer, in Na^+^, 1.5 mM Ca^2+^ saline (middle), and during local perfusion with K^+^, 0.02 mM Ca^2+^ endolymph (bottom). (B) MT currents under perforated-patch in Na^+^, 1.5 mM Ca^2+^ (middle) and during local perfusion with K^+^, 0.02 mM Ca^2+^ endolymph (bottom). Top traces are the bundle stimuli evoked by a piezoelectric-driven glass probe. (C) MT current, I, scaled to its maximum value I_MAX_, versus hair bundle displacement for recording with EGTA. I_MAX_ = 0.90 nA (1.5 Ca^2+^) and 1.45 nA (0.02 Ca^2+^). (D) I/I_MAX_, versus hair bundle displacement for perforated patch recording. I_MAX_ = 0.99 nA (1.5 Ca^2+^) and 1.38 nA (0.02 Ca^2+^) and 0.62 nA (1.5 Ca^2+^ wash). For both conditions, low Ca^2+^ endolymph increased the fraction of MT current on at rest, but this was much smaller with EGTA (0.08) than with perforated patch (0.43).

The fraction of MT current on at rest depended not only on extracellular Ca^2+^ around the hair bundle but also the nature and concentration of the mobile intracellular Ca^2+^ buffer (Ricci et al., 1998; Beurg et al., 2010). BAPTA (1 mM) had been used so far because its properties theoretically match those of the endogenous Ca^2+^ buffer (Beurg et al., 2010), which in OHCs consists of 2 mM oncomodulin plus 0.25 mM calbindin-28K with no significant apex to base gradient (Hackney et al., 2005). To provide experimental support for this, perforated patch recordings were performed on apical OHCs of rats, P9–P11, at which age the oncomodulin concentration is similar to that in the adult (Yang et al., 2004; Hackney et al., 2005). With whole cell recording using 1 mM EGTA (Figures 3A and 3C), exposure to the low Ca^2+^ endolymph increased the MT current amplitude, as with 1 mM BAPTA, but produced only a small change in the fraction of current turned on at rest (mean = 0.12 ± 0.02, n = 5). Under perforated-patch conditions (Figures 3B and 3D), where mobile proteins such as the Ca^2+^ buffers are not washed out, the mean MT channel open resting probability in five OHCs increased from 0.04 ± 0.02 in 1.5 mM Ca^2+^ to 0.42 ± 0.03 in 0.02 mM Ca^2+^. The values obtained with perforated patch did not differ significantly from those obtained in low Ca^2+^ using whole-cell with 1mM intracellular BAPTA in response to either fluid jet (0.43 ± 0.03; Figure 2D) or step stimuli (0.40 ± 0.08) (Beurg et al., 2010). The latter method of hair bundle stimulation also allowed estimates of the adaptation time constant that, as reported previously (Beurg et al., 2010), were slowed in the low Ca^2+^ endolymph and were 0.6 ± 0.03 ms (EGTA), 0.5 ± 0.05 ms (BAPTA), and 1.4 ± 0.4 ms (perforated patch). The slower time constant in perforated patch may largely reflect a greater series resistance (see Experimental Procedures).

### Current Clamp Recordings

**Figure 4:**
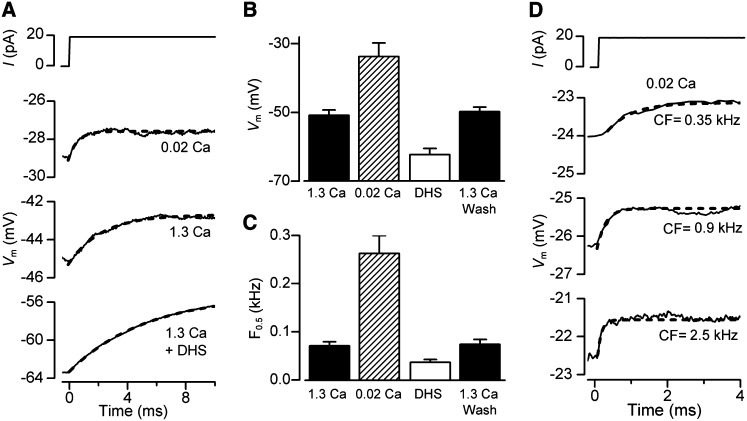
Resting Potentials and Membrane Time Constants (τ_m_) in Gerbil OHCs (A) Voltage responses to current steps in an apical OHC with the hair bundle exposed to 1.3 mM Ca^2+^, 0.02 mM Ca^2+^, and 0.2 mM DHS + 1.3 mM Ca^2+^. Note low Ca^2+^ depolarizes the OHC and reduces τ_m_. Blocking MT channels with DHS hyperpolarizes the OHC and increases τ_m_. τ_m_ was obtained by fitting voltage onsets (dashed lines) with: V = A(1 − exp (−t/τ_m_)), where τ_m_ = 2.3 ms (1.3 Ca^2+^), 0.6 ms (0.02 Ca^2+^), 5.3 ms (1.3 Ca^2+^ + DHS). (B) Collected resting potentials (mean ± SEM) from apical OHCs with bundles exposed to 1.3 mM Ca^2+^ (control, n = 15; wash, n = 14), 0.02 mM Ca^2+^ (n = 5), and 0.2 mM DHS + 1.3 mM or 0.02 mM Ca^2+^ (n = 9). (C) Collected corner frequencies (*F_0.5_* = 1/2πτ_m_; mean ± SEM) calculated from τ_m_ measurements as in (A), numbers of measurements in each category as in (B). (D) Voltage responses to current steps with hair bundles perfused with 0.02 mM Ca^2+^ for three gerbil cochlear locations with different CFs. τ_m_ obtained from fits (dashed lines) and CF: 0.67 ms (0.35 kHz); 0.23 ms (0.9 kHz); 0.10 ms (2.5 kHz).

**Figure 5:**
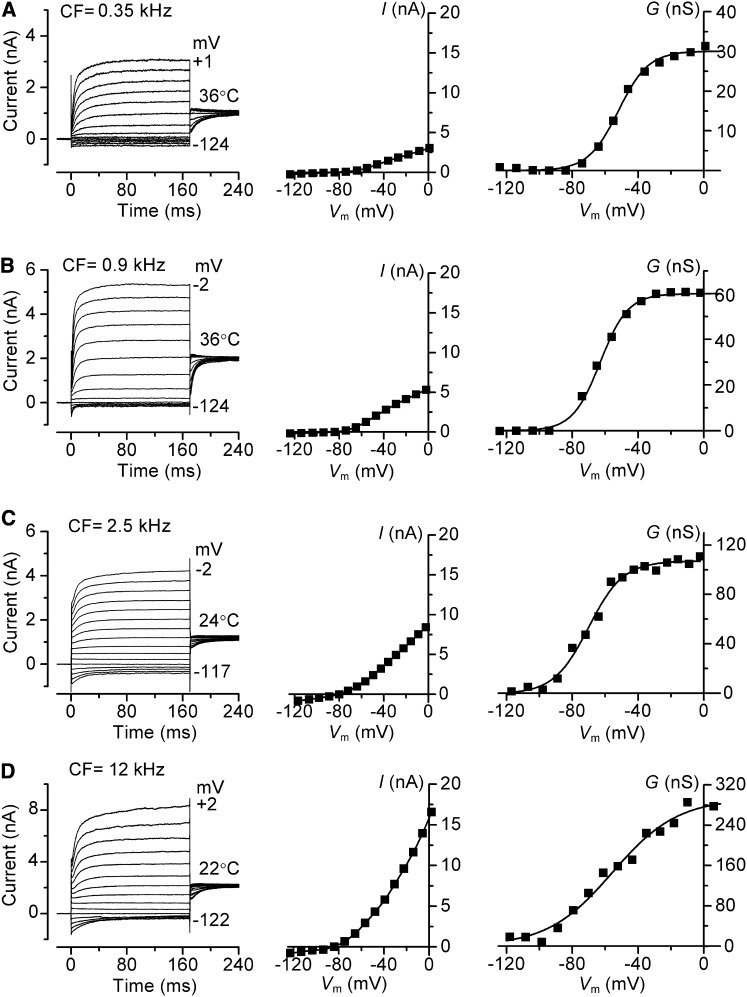
Voltage-Dependent K^+^ Currents in Gerbil OHCs Current records for voltage steps (left), steady-state current-voltage relationships (middle), and conductance-voltage relationships (right) in: P16–P28 animals. CF and recording temperature in (A–D) (left panels) are shown next to traces. Currents were recorded by applying hyperpolarizing and depolarizing voltage steps in 10 mV nominal increments from the holding potential of −84 mV. Holding currents, plotted as zero current, were −148 pA (0.35 kHz), −175 pA (0.9 kHz), −441 pA (2.5 kHz), and −2484 pA (12 kHz).The current-voltage and conductance-voltage relations in (C) and (D) were corrected to 36°C using Q_10_ of 1.7. Conductance G_K_ determined from G_K_ = I/(V − E_K_) where E_K_ is the current reversal potential, −75 mV. G_K_-V relations fitted with single Boltzmann with G_K_ = G_K, MAX_ /(1 + exp (− (V − V_0.5_)/V_S_)) where G_K, MAX_, V_0.5_, V_S_ are (A) 30 nS, −52 mV, 9.2 mV; (B) 60 nS, −63 mV, 8.4 mV; (C) 107 nS, −67 mV, 11 mV; (D) 280 nS, −56 mV, 18 mV.

**Figure 6:**
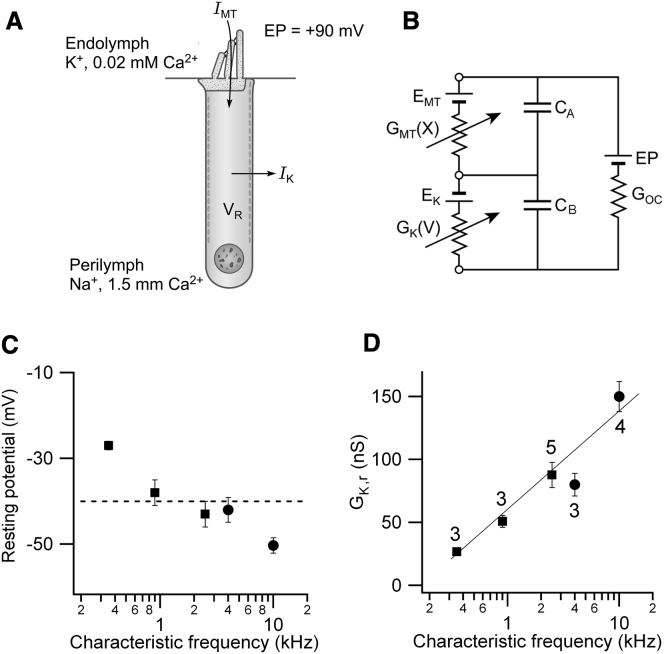
Predicted In Vivo OHC Resting Potentials (A) Two main ionic currents in OHCs, I_MT_ flowing through MT channels in the hair bundle that faces endolymph and I_K_ exiting through voltage-dependent K^+^ channels in the basolateral membrane facing perilymph. There is a +90 mV potential (EP) between endolymph and perilymph. (B) Equivalent electrical circuit for the OHC containing MT conductance, G_MT_(X), modulated by bundle displacement X and in series with battery E_MT_, the reversal potential for the MT channels (0 mV); and G_K_(V) gated by membrane potential V in series with battery E_K_, the reversal potential for the K^+^ channels (−75 mV). G_OC_ is a parallel organ of Corti conductance, large compared to OHC membrane conductances. The capacitances of the apical (C_A_) and basolateral (C_B_) membranes are also indicated. (C) Predicted resting potential, V_R_ (mean ± SEM), versus CF calculated by inserting into circuit measured values of G_MT_ and G_K_, corrected to T = 36°C. Dashed line, mean of all values = −40 mV. (D) K^+^ conductance (G_K,r_) at OHC resting potential (mean ± SEM) versus CF. Number of measurements indicated beside each point.

**Figure 7:**
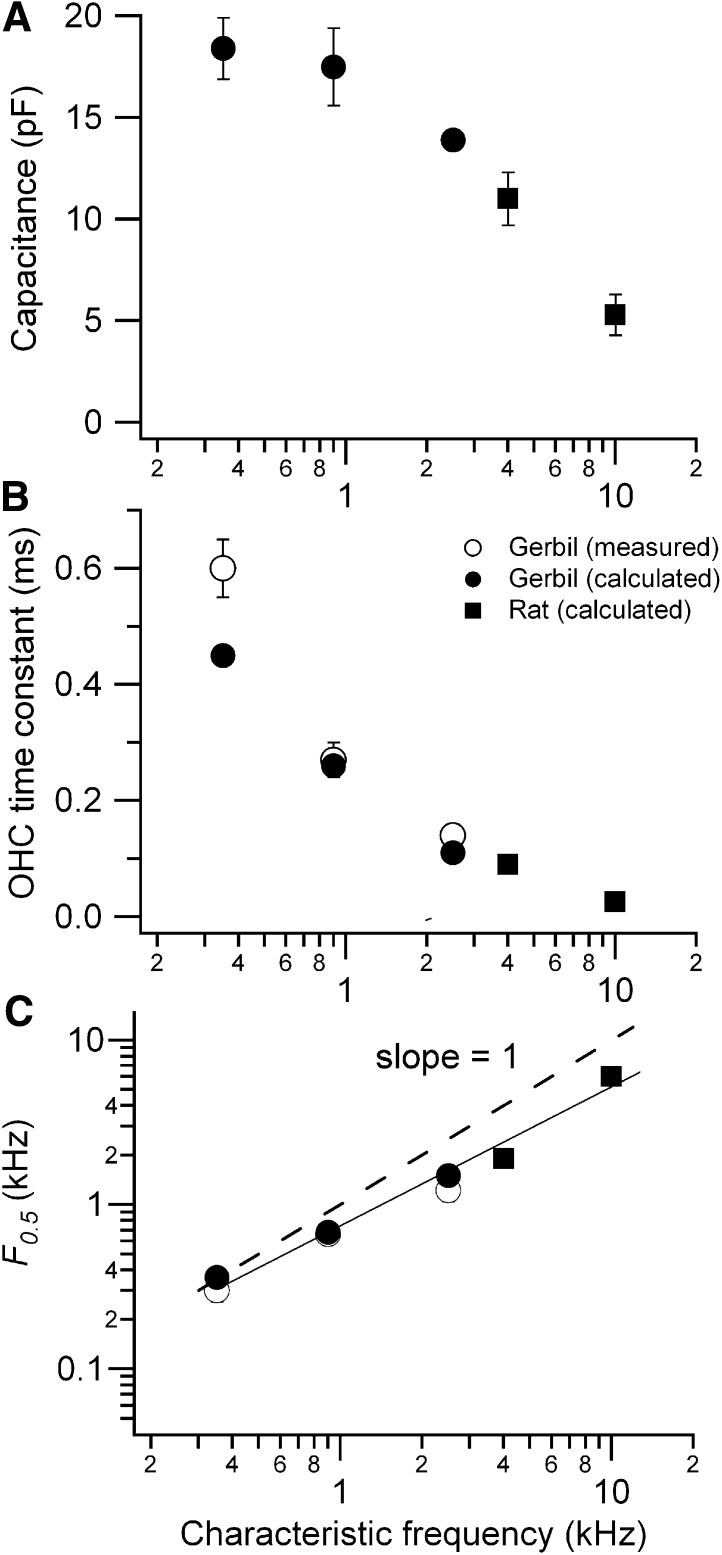
Predicted In Vivo Membrane Time Constant and Corner Frequency (A) Linear OHC membrane capacitance, C_m_, (mean ± SEM) versus CF for gerbils (filled squares) and rats (filled circles); (B) τ_m_ (mean ± SEM) versus CF for gerbils (open squares from Figure 4; filled squares, predicted) and rats (filled circles, predicted); (C) corner frequency, F_0.5_ (= 1/2πτ_m_) versus CF; continuous line is the fit to the data and the dashed line has slope of 1, where CF equals F_0.5_.

**Figure 8:**
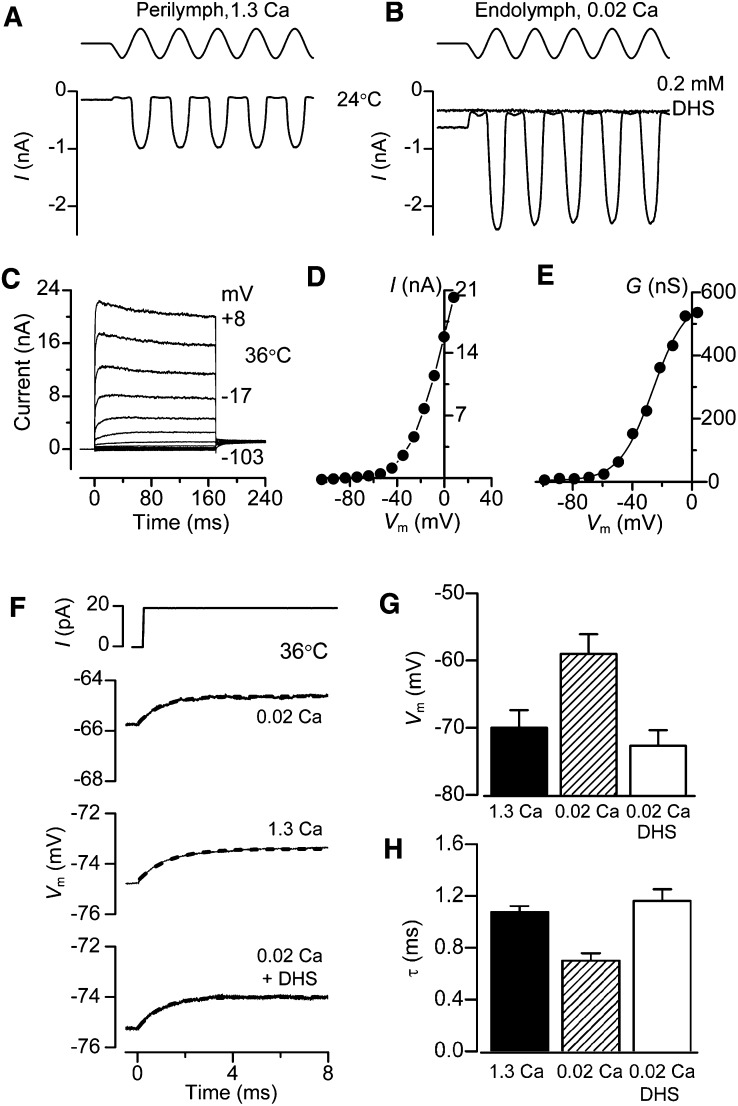
Membrane Currents, Resting Potentials, and τ_m_ in Gerbil IHCs (A and B) Saturating MT currents in apical-coil gerbil IHCs exposed to 1.3 mM Ca^2+^ (A) and endolymphatic Ca^2+^ (0.02 mM) (B). Note the larger MT current amplitude and increased P_OPEN_ at rest compared to 1.3 mM Ca^2+^. In 0.02 mM Ca^2+^ ∼20% of the maximum MT current was activated at rest and this was abolished by 0.2 mM DHS, T = 24°C. (C–E) Voltage-dependent K^+^ currents in apical gerbil IHCs. Current records for voltage steps in 10 mV nominal increments (C), steady-state current-voltage (D), and conductance-voltage relationships (E) in a P18 IHC, T = 36°C. Conductance G_K_ was determined as described for OHCs (Figure 5) and the conductance-voltage relation was fitted with a single Boltzmann where G_K, MAX_, V_0.5_, V_S_ are: 580 nS, −26 mV, 11 mV. (F) Voltage responses to current steps in apical IHCs with hair bundle exposed to 1.3 mM Ca^2+^, 0.02 mM Ca^2+^, and 0.2 mM DHS + 0.02 mM Ca^2+^. τ_m_ from fitting voltage onsets (dashed lines): 1.0 ms (1.3 Ca^2+^), 0.8 ms (0.02 Ca^2+^), 1.0 ms (0.02 Ca^2+^ + DHS). (G) IHC resting potential (mean ± SEM; n = 4) for the three conditions in (F). (H) Time constant τ_m_ (mean ± SEM) from recordings as in (F).

In order to measure the effects of low endolymphatic Ca^2+^ on membrane time constant and resting potential, current clamp experiments were performed at body temperature on OHCs from isolated gerbil cochleas at around the onset of hearing (P11–P13). These experiments reinforced the findings that exposing the hair bundles and transduction apparatus to low (0.02 mM) Ca^2+^ caused a depolarization of the OHC (Figures 4A and 4B) to −34 ± 4 mV (n = 5). The procedure was fully reversible on returning to high (1.3 mM) Ca^2+^, excluding the possibility that the observed changes in membrane potential were due to cell deterioration during the course of the recordings. The resting potential in the presence of 0.2 mM DHS, the MT channel blocker, was more hyperpolarized (−62 ± 2 mV, n = 9) than in high Ca^2+^ (−50 ± 2 mV, n = 15: Figure 4B), suggesting that the small fraction of channels open at rest (0.08: from gerbil OHCs) in high Ca^2+^ was sufficient to evoke some cell depolarization. The resting membrane potential in the presence of DHS was not significantly different from that obtained when MET channels were mechanically shut off (−61.3 ± 2.0, n = 5, Figures 2A and 2B) or when hair bundles were superfused with a Ca^2+^-free solution (−63.5 ± 2.0, n = 5), a condition that abolishes the MET current (Crawford et al., 1991). This further supports our hypothesis that the depolarized membrane potential of OHCs results from the resting MET current and not a nonspecific leak.

A consequence of the depolarized resting potential in low Ca^2+^ was a reduction in the membrane time constant (τ_m_) that was derived from current-clamp recordings of the voltage responses (V) to a small current step (I) at around the resting membrane potential. The time course of the voltage onsets can be described by: V = IR_m_(1 − exp (−t/τ_m_)) where R_m_ is the membrane resistance and t is time. τ_m_ was derived from fits to these onsets (Figure 4A), and at the cochlear apex, was found to decrease from 2.2 ± 0.3 ms (n = 15) in 1.3 mM Ca^2+^ to 0.6 ± 0.1 ms (n = 5) in 0.02 mM Ca^2+^. When the MT channels were blocked with 0.2 mM DHS, the resting potential hyperpolarized and τ_m_ increased to 5.3 ± 0.1 ms (n = 9). The membrane time constant behaves like a single-pole low-pass filter with a half-power or corner frequency, *F_0.5_*, equal to 1/2πτ_m_. Thus factors that reduce τ_m_ increase *F_0.5_* and expand the frequency range at which OHCs can operate; lowering the Ca^2+^ concentration in the solution bathing the hair bundle reduces τ_m_ and increases *F_0.5_* whereas blocking the MT channels increases τ_m_ and lowers *F_0.5_* (Figure 4C). Strikingly, when OHCs were examined in low Ca^2+^ at other cochlear locations with higher CFs, the voltage responses to current steps had faster onsets and smaller values for τ_m_, resulting in higher corner frequencies (Figure 4D). This observation suggests that the corner frequency of the OHC membrane varies with the CF of the cell.

### The In Vivo Resting Potential and Corner Frequency

The results so far were obtained in isolated preparations in which the conditions still differ from those in vivo. Although account has been taken of the endolymph composition and the temperature, there exists in vivo a 90 mV potential (Bosher and Warren, 1971) across the hair cell epithelium between the endolymphatic and perilymphatic compartments. An endolymphatic potential will augment the driving force on current flow through the MT channels and aid with depolarization. Furthermore, although the MT current has attained its full size prior to the onset of hearing (Kennedy et al., 2003; Waguespack et al., 2007), the voltage-dependent K^+^ current continues to increase during the third postnatal week as the endolymphatic potential attains its mature value (Bosher and Warren, 1971). The voltage-dependent K^+^ currents were measured in older (P16–P28) animals (Figure 5), an age range where the size of the K^+^ current has reached its fully mature level (Marcotti and Kros, 1999). The predominant current in adult OHCs is a negatively activated delayed rectifier K^+^ current named I_K,n_ (guinea-pig, Mammano and Ashmore [1996]; mouse, Marcotti and Kros [1999]) flowing through channels containing KCNQ4 subunits (Kubisch et al., 1999; Kharkovets et al., 2006). The relaxation of the current at negative potentials and the observation that it could be blocked by 20 μM XE991 (data not shown), a blocker of KCNQ channels (Kharkovets et al., 2006), suggest the K^+^ currents in both rats and gerbils are also dominated by I_K,n_. However, the contribution of I_K,n_ to the total K^+^ current increased as a function of OHC position along the cochlea, with an apex to base gradient (Figure 5), as previously shown in the guinea-pig (Mammano and Ashmore, 1996). The K^+^ conductance was activated at negative membrane potentials (gerbil, V_0.5_ = −62 ± 3 mV, n = 15; rat, V_0.5_ = −74 ± 7 mV, n = 7), was almost saturated at −30 mV (Figure 5) and its maximum value increased along the tonotopic axis in both gerbil (∼9-fold for CFs 0.35–12 kHz: Figures 5A–5D) and in rat (∼2-fold for CFs 4–10 kHz; data not shown). The maximum K^+^ conductances at different CFs, corrected to 36°C (see Experimental Procedures), were, for gerbils, 29 ± 1 nS (n = 3) at 0.35 kHz; 57 ± 5 nS (n = 3) at 0.9 kHz; 90 ± 10 nS (n = 5) at 2.5 kHz and 256 ± 36 nS (n = 4) at 12 kHz; and for rats, 85 ± 12 nS (n = 3) at 4 kHz and 241 ± 30 nS (n = 4) at 10 kHz (see Figure S1A available online).

The resting potentials in vivo will be determined by the balance between the standing inward current through the MT channels and the outward current via the voltage-dependent K^+^ channels (Figure 6A). The theoretical in vivo resting potential can be calculated from a simple electrical circuit for the OHC (Figure 6B) (Dallos, 1985b). The circuit includes the MT conductance, G_MT_(X), in the hair bundle, gated by hair bundle displacement X, and the voltage-dependent K^+^ conductance, G_K_(V), in the OHC basolateral membrane that is in series with a battery (E_K_) representing the reversal potential for the K^+^ channels (−75 mV) (Marcotti and Kros, 1999). A battery (E_MT_) has also been added (Figure 6B) to represent the reversal potential of the MT channels but measurements indicate this is approximately zero millivolts (Kros et al., 1992; Beurg et al., 2006) so it will be ignored. The electrical driving force for the standing MT current comprises the resting potential (V_R_) and an endolymphatic potential (EP) of +90 mV (Bosher and Warren, 1971). The organ of Corti conductance (G_OC_) is ignored because it is several orders of magnitude larger than the OHC membrane conductances (Dallos, 1983). For completeness, the membrane capacitance was also included in Figure 6B, but in the steady state, the electrical circuit is described by:

(1) $${\text{G}}_{\text{MT},\text{r}}\;\left(90-{\text{V}}_{\text{R}}\right){=\text{G}}_{\text{K},\text{r}}\;\left({\text{V}}_{\text{R}}\;-{\text{E}}_{\text{K}}\right),$$

Because G_K_(V) varies monotonically with membrane potential, Equation 1 can be used to obtain a unique solution for V_R_ derivable by iteration. Measured values for the resting MT conductance, G_MT,r_, and the K^+^ conductance (G_K,r_) at the resting potential were corrected, where necessary, to 36°C, close to body temperature, using measured Q_10_ coefficients (see Experimental Procedures). The calculations were performed for the five CFs, corresponding to three gerbil and two rat cochlear locations and predicted an overall resting potential of −40 ± 4 mV (n = 18). The trend of increasingly hyperpolarized resting potential with CF from about −30 to −50 mV (Figure 6C**)** reflects the larger tonotopic gradient in the amplitude of the K^+^ conductance compared to that of the MT conductance. The K^+^ conductance at this resting potential increased monotonically with CF to offset the tonotopic increase in the MT conductance (Figure 6D). Therefore taking account of the fully developed K^+^ conductance and the endolymphatic potential, the predicted resting potential is not very different from that measured in younger animals (Figure 4B). At this resting potential, the voltage-dependent K^+^ conductance was almost fully activated.

Knowing the OHC total membrane conductance G_r_ at the resting potential (G_r_ = G_MT,r_ + G_K,r_), it is now possible to calculate the membrane time constant (τ_m_ = C_m_/G_r_) where C_m_ is the total membrane capacitance (C_m_ = C_A_ + C_B_; Figure 7A). The calculations demonstrate that τ_m_ declines from about 0.6 ms to 25 μs with an increase of CF from 0.35 to 10 kHz (Figure 7B). This tonotopic variation stems from a reduction in the linear capacitance, attributable to shorter OHCs, and an increase in membrane conductance due to the tonotopic gradients in both G_MT_ and G_K_. As a consequence of the change in τ_m_ , *F_0.5_*, the OHC corner frequency, increases with CF, roughly matching it (Figure 7C). As the CF changes from 0.35 to 10 kHz, the corner frequency increases from 0.3 to 6.4 kHz. The slope of the relationship is, however, less than unity (the dashed line in Figure 7C). The deviation from unity slope is most easily explained by the maximum MT current being under estimated in cells tuned to higher CFs, because of damage to or rapid deterioration of such OHCs during isolation. The same problem may account for the increasingly negative predicted resting potentials at the higher CFs (Figure 6C). These factors have also precluded study of the most basal cells.

The filtering imposed by the membrane RC time constant during modulation of the MT conductance will be greater than that observed during extrinsic current injection because the capacitance of the hair bundle bearing membrane (C_A_ in Figure 6) will partially shunt the MT current. The degree of shunting depends on the proportion of the capacitance contributed by C_A_ (if C_A_ = 0 no shunting will occur). To address this, the areas of the apical and basolateral membranes were estimated from the dimensions of rat OHCs and their hair bundles (Roth and Bruns, 1992; Beurg et al., 2006) yielding a C_A_/C_B_ ratio of 0.20 independent of CF. The areas of the endolymphatic (hair bundle plus apical membrane) and perilymphatic membranes are: 333 μm^2^, 1650 μm^2^ (low CF); 135 μm^2^, 678 μm^2^ (mid CF); 79 μm^2^, 390 μm^2^ (high CF). This surprising result stems from a 5-fold reduction in stereociliary height (average height, 4–0.8 μm, assumed as half the maximum height) and diameter (0.25–0.15 μm; D. Furness, personal communication), which reduces C_A_, along with a decrease in OHC length (50 to 16 μm) and diameter (10 to 7 μm) contributing to C_B_. Linear analysis of the circuit (Figure 6B) was performed using these capacitance values by calculating the receptor potential amplitude for a 10% modulation in MT conductance at CFs from 0.3 to 10 kHz. With increasing CF, the receptor potential was reduced from 3.6 to 1.9 mV (C_A_/C_B_ = 0.2) compared to 4 to 2.1 mV (C_A_ = 0). The difference between these two sets of values is about 15%, suggesting the apical area has been reduced to minimize shunting of the MT current.

### Inner Hair Cells

In order to verify whether the effects of endolymphatic Ca^2+^ on the MT channel, resting membrane potential, and time constant were specific to OHCs, we performed experiments on inner hair cells (IHCs) that lack prestin (Zheng et al., 2000) and have the principal role of synaptically transmitting the auditory signal to spiral ganglion cell dendrites. In contrast to OHCs, there is no evidence of tonotopic variation in either the MT conductance (Beurg et al., 2006; Jia et al., 2007) or the voltage-dependent K^+^ conductance (Kimitsuki et al., 2003; Marcotti et al., 2003). Furthermore, compared to OHCs, IHCs have a tenth the concentration of proteinaceous Ca^2+^ buffer (Hackney et al., 2005), which was previously assessed from perforated-patch recordings as equivalent to 1 mM EGTA (Johnson et al., 2008). To determine the IHC parameters, measurements were made on gerbil apical IHCs with electrodes containing 1 mM EGTA (see Experimental Procedures). As with OHCs, perfusing 0.02 mM Ca^2+^ increased the peak size of the MT current and also the fraction activated at rest (Figures 8A and 8B). The mean MT current increased from 0.79 ± 0.07 nA (1.3 Ca^2+^; n = 4) to 1.72 ± 0.12 nA (0.02 Ca^2+^; n = 5, T = 23°C) and the fraction on at rest increased from 0.045 ± 0.004 (1.3 Ca^2+^) to 0.17 ± 0.03 (0.02 Ca^2+^). Using the latter fraction and correcting the standing current to 36°C yields a resting MT conductance of 5.7 nS. Because the voltage-dependent K^+^ conductance changes with development (Marcotti et al., 2003), its adult value was measured in P18 animals (Figures 8C–8E). The conductance-voltage relationships could be fit with a single Boltzmann (Figure 8E) with G_MAX_ = 470 ± 96 nS, V_0.5_ = −31 ± 3 mV and V_S_ = 10.5 ± 3.5 mV (n = 5). The K^+^ conductance is larger than in OHCs and when combined with the smaller standing MT conductance suggests a more hyperpolarized resting potential than in OHCs. The resting potential was determined in two ways as for OHCs. During current clamp recordings in isolated cochleas of P18 animals (Figures 8F–8H), perfusing 0.02 mM Ca^2+^ depolarized the IHC from −70 ± 3 mV (n = 4) to −59 ± 3 mV and reduced the membrane time constant from 1.08 ± 0.05 ms in 1.3 mM Ca^2+^ to 0.70 ± 0.06 ms in 0.02 mM Ca^2+^. A second method was to apply Equation 1, using 5.7 nS for the resting MT conductance, and determining which membrane potential, V_R_, satisfied the equation for each of the measured G_K_-V relationships; E_K_ was assumed to be −75 mV. This calculation improves on the direct recording by taking into account the endolymphatic potential and thus predicting IHC properties in vivo. The resting potential was calculated as −55 ± 2 mV (n = 5) comparable to that obtained by direct measurement in the isolated cochlea. With the measured IHC capacitance (12.5 ± 0.5 pF), the membrane time constant was 0.26 ± 0.03 ms (n = 5), equivalent to a corner frequency of 0.61 kHz, which is similar to that found in vivo (Palmer and Russell, 1986).

## Discussion

The difficulties of recording from and directly stimulating OHCs in the in vivo cochlea has motivated work on isolated pieces of the organ of Corti or cochlear slices in which large transduction currents can be obtained from single hair cells (Kros et al., 1992; Kennedy et al., 2003; He et al., 2004). However, because the organ of Corti is a tight epithelium dividing two fluid compartments with distinct ionic compositions, use of isolated preparations has the drawback that the environmental conditions usually differ from those in vivo: the hair bundles are not exposed to endolymph containing low, 20–40 μM, Ca^2+^, the 90 mV endolymphatic potential across the epithelium is absent and, to prolong the viability of the preparation, measurements are mostly made at room temperature. We therefore corrected for these differences with the justification that OHC MT currents obtained in isolated preparations of younger animals (P7–P13) are the best currently achievable (Kennedy et al., 2003). Our results showed that OHCs have a relatively depolarized resting potential (−30 to −40 mV), based both on direct current-clamp measurements near body temperature in animals around the onset of hearing (P11–P13), and from extrapolations to the mature in vivo condition (P16–P19). The agreement between the direct measurements and the predictions may be fortuitous, because the latter incorporate an endolymphatic potential and values for a near-fully developed K^+^ conductance in older P16–P19 animals. However, these two factors will tend to balance each other out as the development of the endolymphatic potential will increase the MT current to offset the growth in the K^+^ conductance over the same period (P11–P19).

It might be argued that the standing MT current and the depolarization elicited during bundle perfusion with low Ca^2+^ solution are an artifact of the local perfusion system, perhaps due to damage to the OHCs or exposure of the basolateral membrane to high K^+^. This seems unlikely for the following reasons: (1), any nonspecific leak current or depolarization could be abolished with 0.2 mM DHS (Figure 2 and Figure 4) that blocks the MT channel without affecting the voltage-sensitive K^+^ current; indeed perfusion with DHS was used to define the nontransducer dependent leak current; (2), OHCs showed a hyperpolarized resting membrane potential (negative to −60 mV) in conditions that turned off the MET current, which indicates the presence of healthy cells; (3), comparison of the fraction of MT current on at rest in rat and gerbil gave the same value (0.46) irrespective of whether the low Ca^2+^ endolymph was accompanied by K^+^ or Na^+^, which have similar permeability through the MT channel (Ohmori, 1985); furthermore, membrane potentials in gerbil OHCs (Figure 4) were measured with a Na^+^-based endolymph; and (4), the standing current, however, relied on the nature of the intracellular mobile Ca^2+^ buffer and was smaller with EGTA than with BAPTA (Figure 3). The distinction between BAPTA and EGTA largely reflects a difference in the rate of Ca^2+^ binding, BAPTA being much faster in lowering the Ca^2+^ near the internal face of the MT channel (Ricci et al., 1998). This accounts for the difference in the fraction of MT channels open at rest and in resting potential between OHCs (endogenous Ca^2+^ buffer equivalent to 1 mM BAPTA; Beurg et al., 2010) and IHCs (1 mM EGTA; Johnson et al., 2008).

The OHC resting potentials in endolymphatic Ca^2+^ reported here differ from earlier measurements using other types of preparation and experimental conditions. Most studies on isolated organs of Corti or solitary OHCs have reported resting potentials of −60 to −70 mV (e.g., −57 mV, Housley and Ashmore, 1992; −64 mV, Preyer et al., 1994; −70 mV, Mammano and Ashmore, 1996; −60 mV, Marcotti and Kros, 1999). In those recordings, receptor potentials were only a few millivolts (Preyer et al., 1994) or not reported, suggesting a small standing MT current, a view supported by the more hyperpolarized membrane potential of OHCs obtained on turning off the MET current (Figures 2 and 4) or tip link destruction. The preparation most similar to that used here is the hemi-cochlea (He et al., 2004), which gave a mean OHC resting potential of −57 mV and a maximum receptor potential of 30 mV in the presence of 1.6 mM extracellular Ca^2+^. These values were similar to our measurements when the entire preparation was bathed in high Ca^2+^ saline (V_R_ = −51 mV). In vivo recordings using sharp electrodes have given resting potentials for OHCs of −70 to −83 mV and receptor potentials were generally <15 mV (Dallos, 1985a; Russell et al., 1986), although isolated examples of 30 mV (Dallos, 1986) and 34 mV (Russell and Kössl, 1992) have been reported. However, the disagreement may be less than it appears because Dallos (1985a, 1985b) noted that the resting potential of apical OHCs immediately after cell penetration had a median value of −55 mV but then shifted negative to about −70 mV, the hyperpolarization often being accompanied by a reduction in receptor potential amplitude. The OHC with largest receptor potential in Russell and Kössl (1992) also had a low resting potential of −52 mV compared to the population mean. The simplest interpretation is that hyperpolarization is attributable to loss of mechanotransduction. The receptor potentials measured in vivo were several-fold smaller than those we obtained (Figures 1 and 2), implying an equivalent reduction in the standing inward transducer current in vivo such that OHCs were likely to be more hyperpolarized. We suggest that OHC resting potentials of −70 mV may not accurately reflect the in vivo situation but instead indicate, for whatever reason, a decrease in the MT current and loss of the resting inward current.

An advantage of having the MT channels half-activated at rest is that the OHC receptor potentials to tonal stimuli will remain approximately sinusoidal with increasing intensity; if the resting open probability is small, as with the IHCs, the response will become rectified with voltage excursions on the positive half of the cycle being much larger than on the negative half. These differences in response waveform between the two types of hair cell were observed in vivo (Russell et al., 1986) and may be manifested in the extracellularly-recorded potentials thought to reflect the MT currents. Thus the cochlear microphonic (the periodic component) may arise predominantly from the OHCs and the summating potential (the DC component) from the IHCs. The difference in resting potentials between the types of hair cell may also be linked to optimizing their disparate functions, cochlear amplification in OHCs, and synaptic transmission in IHCs. By analogy, a standing inward MT current depolarizes turtle auditory hair cell to −45 mV, near the membrane potential at which electrical tuning is maximal (Farris et al., 2006). The OHC resting potential of −40 mV may be similar to the membrane potential where prestin has the steepest voltage sensitivity. In OHCs of rats with fully developed hearing, the half-activation voltage for prestin has been reported as about −40 mV (Oliver and Fakler, 1999; Mahendrasingam et al., 2010). However, measurements in other preparations have found more hyperpolarized values (reviewed in Ashmore, 2008) that depend on various environmental factors including membrane tension (Kakehata and Santos-Sacchi, 1995), intracellular chloride (Rybalchenko and Santos-Sacchi, 2003), and phosphorylation (Frolenkov et al., 2000). Resolving the exact value may require determining the prestin half-activation voltage in vivo. In contrast, the predicted IHC resting potential of −55 mV is near the membrane potential at which the voltage-dependent Ca^2+^ current mediating synaptic transmission begins to activate at body temperature (−60 mV; Grant and Fuchs, 2008; Johnson and Marcotti, 2008).

The main consequence of a depolarized resting potential in OHCs is full activation of the voltage-dependent K^+^ conductance, thus minimizing τ_m_ and expanding the membrane filter so there is little attenuation of CF receptor potentials. Previous estimates of OHC τ_m_ translate into equivalent corner frequencies an order of magnitude less than the CF (Mammano and Ashmore, 1996; Preyer et al., 1994, 1996). For example, corner frequencies of 15, 50, and 480 Hz were measured in turns 4 (CF = 0.5 kHz), 3 (CF = 2 kHz), and 2 (CF = 7 kHz) of the guinea pig cochlea (Mammano and Ashmore, 1996), but this is unsurprising, as the OHCs had resting potentials of −70 mV where the K^+^ conductance would be only partially activated. For turns 3 and 4, these lower corner frequencies are similar to the ones measured here when the MT channels of apical gerbil OHCs (CF = 0.35 kHz) were blocked with DHS (about 40 Hz; Figure 4C). Our results demonstrate a similarity between the membrane corner frequency and CF (Figure 7), and if this extends to even higher frequencies, the amplitude of CF receptor potentials will be not greatly attenuated over the entire auditory range. This property removes a major criticism for the contribution of prestin-induced somatic contractility to the cochlear amplifier. To examine the extension to the highest frequencies, the tonotopic gradients were extrapolated to the upper frequency limit in the rat (55 kHz), giving 700 nS and 130 nS for the K^+^ and MT conductances, respectively (Figure S1). Using these values and a membrane capacitance of 4.5 pF, a resting potential of −53 mV and a corner frequency of 18 kHz were inferred. As noted earlier, the imperfect agreement between the CF and corner frequency may in part stem from the MT current being underestimated in our experiments. Nevertheless, the approximate match over much of the frequency range ensures activation of prestin by receptor potentials at CF facilitating its role in cochlear amplification. More work is needed to determine whether other mechanisms, such as extracellular potential fields (Mistrík et al., 2009), also contribute at the highest CFs.

## Experimental Procedures

Recordings were made from OHCs in isolated organs of Corti of Sprague-Dawley rats and Mongolian gerbils between 6 and 28 days postnatal (P6–P28, where P0 is the birth date) and IHCs from gerbils (P8 and P18) using methods previously described (Kennedy et al., 2003; Marcotti et al., 2005; Johnson and Marcotti, 2008).

### Rat Preparation and Recordings

Rats were killed by decapitation for P8–P11 animals, but older ones (P15–P19) were first anesthetized with halothane prior to decapitation using methods approved by the Institutional Animal Care and Use Committee of the University of Wisconsin-Madison according to current National Institute of Health guidelines. Excised apical or middle cochlear turns were viewed through a water-immersion objective (Zeiss 40× or 63×) on a Zeiss Axioskop FS microscope. The chamber was perfused with artificial perilymph of composition (in mM): 150 NaCl, 6 KCl, 1.5 CaCl_2_, 2 Na-pyruvate, 8 D-glucose, and 10 Na-HEPES (pH 7.4), osmolarity 315 mOsm/kg^−1^. The effect of endolymph was examined by changing the solution around the hair bundle using a nearby puffer pipette to one containing (mM): 155 KCl, 0.02 CaCl_2_ (buffered with 4 HEDTA), 2 Na-pyruvate, 8 D-glucose, and 10 K-HEPES (pH 7.4). Endolymph Ca^2+^ has been reported to be between 0.02 and 0.04 mM (Bosher and Warren, 1978; Salt et al., 1989). The puffer pipette was positioned about 30 μm from the target and aimed approximately along the cochlear axis so the flow did not directly stimulate the bundle. The flow was also away from the small hole in the reticular lamina through which the recording electrode was introduced so it is unlikely that the solution gained access to the OHC's basolateral membrane. To ensure that the solution was fully replaced, the flow was continued until the holding current had increased to a steady state (usually taking 10–20 s) prior to running the stimulation protocol. Recordings were made from first or second row OHCs using borosilicate patch electrodes connected to an Axopatch 200A amplifier and currents were low-pass filtered at the amplifier output at 10 kHz and digitized at 100 kHz. Patch electrodes were filled with an intracellular solution containing (mM): 125 KCl, 3.5 MgCl_2_, 5 Na_2_ATP, 0.5 GTP, 10 Tris phosphocreatine, 1 BAPTA, 10 K-HEPES (pH 7.2), osmolarity 295 mOsm/kg^−1^. BAPTA (1 mM) was used as the intracellular Ca^2+^ buffer as it most closely approximates the native buffer (Beurg et al., 2010). No significant apex to base gradient in the Ca^2+^ buffer concentration has been reported (Hackney et al., 2005) so the same BAPTA concentration was used for all CFs. In recording from older (P15–P19) animals, intracellular chloride was reduced to minimize OHC contractions by replacing the 140 KCl with 130 K-aspartate plus 10 KCl. The locations of the apical, middle and a few basal turn recordings (Figure S1) correspond in vivo to mean CFs of 4, 10, and 20 kHz respectively for P21 animals (Müller, 1991). Because there is a continued expansion of the high frequency range into the adult for both rat and gerbil (Müller, 1991; 1996), CFs were taken from frequency maps at P21.

### Gerbil Preparation and Recordings

Gerbils (P6–P28) were killed by cervical dislocation in accordance with UK Home Office regulations and the dissected cochlear turns transferred to a microscope chamber, immobilized under a nylon mesh attached to a stainless steel ring, and continuously perfused with normal extracellular solution (mM): 135 NaCl, 5.8 KCl, 1.3 CaCl_2_, 0.9 MgCl_2_, 0.7 NaH_2_PO_4_, 5.6 d-glucose, 2 Na-pyruvate, 10 Na-HEPES (pH 7.5), osmolarity 308 mOsmol/kg^−1^. The effect of endolymphatic Ca^2+^ concentration (0.02 mM or 0.04 mM CaCl_2_) was examined by superfusing the hair bundle with a solution similar to that used for rats but usually using Na^+^ as the major monovalent cation instead of K^+^. Organs of Corti were viewed on a Leica DMLFS upright microscope (Wetzlar, Germany) equipped with Nomarski optics through a 63× water-immersion objective. Recordings were made from second or third row OHCs and IHCs using soda glass patch pipettes coated with surf wax (Mr Zoggs SexWax, USA) to minimize pipette capacitance. For OHC recordings, pipettes were filled with an intracellular solution containing (mM) 131 KCl, 3 MgCl_2_, 5 MgATP, 10 K_2_-phosphocreatine, 1 BAPTA, 5 K-HEPES (pH 7.3), osmolarity 293 mOsmol/kg^−1^. In some experiments, the KCl was replaced by 110 K Gluconate plus 15 KCl. For IHCs, 1 mM EGTA was used instead of BAPTA in the above intracellular solution. Electrophysiological recordings were made using an Optopatch amplifier (Cairn Research Ltd, UK). Data acquisition was controlled by pClamp software using a Digidata 1440A (Molecular Devices, CA). Depending on the experiment, data were low-pass filtered at 2.5–50 kHz and sampled at 5–200 kHz. Four cochlear locations were assayed in the apical, middle, and basal turns corresponding in vivo to mean CFs of 0.35, 0.9, 2.5, and 12 kHz, respectively at P18 (Müller, 1996). All current clamp experiments were performed at 36°C.

All membrane potentials were corrected for a liquid junction potential (−4 mV for intracellular solution based on KCl, −12 mV for K-gluconate, and −14 mV for K-aspartate) and for the voltage drop across the uncompensated series resistance. For whole cell recordings, electrodes had starting resistances of 1–10 MΩ and with ≤90% compensation, had a residual series resistances of 0.4–4 MΩ and time constants of <45 μs. For K^+^ current recordings, the residual series resistance was 1 MΩ or less. Most voltage-clamp protocols are referred to a holding potential of −84 mV; membrane capacitances were determined at this holding potential by patch-clamp amplifier compensation of the current transient. Values are given as mean ± SEM and p < 0.05 indicates statistical significance on a two-tailed Student's t test.

### Perforated Patch Recordings

To examine the contribution of the cytoplasmic Ca^2+^ buffer, some experiments were performed using whole-cell recordings with 1 mM EGTA instead of BAPTA and also under nystatin perforated-patch conditions where the mobile endogenous calcium buffer is retained in the cell (Ricci et al., 1998). For perforated patch recordings, the pipette solution contained (mM): 135 K-aspartate, 10 KCl, 5 MgATP, 1 EGTA, 10 K-HEPES (pH 7.2) with or without nystatin; 2.4 mg of nystatin (Calbiochem, San Diego, CA) was dissolved in 10 μl of dry dimethylsulfoxide and diluted 1:500 into the pipette solution. The patch pipette was tip-filled with antibiotic-free stock solution and back-filled with the nystatin solution. Series resistances in perforated-patch mode were 11–24 MΩ (mean ± SD, 17 ± 6 MΩ) with no compensation and mean recording time constants of 100 ± 50 μs. For these experiments, hair bundle stimulation was implemented with a fire-polished glass pipette attached to a piezoelectric stack actuator (PA8/12, Piezosystem Jena) (Kennedy et al., 2003). Perforated patch recordings were performed on rats of P9–P11, at which age the major buffer oncomodulin (parvalbumin β) is close to its adult concentration (Yang et al., 2004; Hackney et al., 2005).

### Hair Bundle Stimulation

In both rat and gerbil experiments, hair bundles were usually mechanically stimulated by a fluid jet from a pipette, tip diameter 5−10 μm driven by a 25 mm diameter piezoelectric disc as previously documented (Kros et al., 1992). The distance of the pipette tip from the bundle was adjusted to elicit a maximal MT current. Saturating mechanical stimuli were applied as 40 or 50 Hz sinusoids with driving voltage of ∼40 V peak-to-peak. When testing the effects of endolymph, the fluid jet pipette was normally filled with a solution containing low (0.02 mM) Ca^2+^. During application of a mechanical stimulus, the fluid around the hair bundle was also exchanged for the same low Ca^2+^ solution. Bundle motion during fluid jet stimulation was determined in rat experiments by projecting an image of the OHC bundle onto a pair of photodiodes (LD 2-5; Centronics, Newbury Park, CA) at 340× total magnification (Kennedy et al., 2006). The differential photocurrent was filtered at 5 kHz. It was calibrated by measuring its amplitude when displacing the photodiodes a known amount in the image plane then using the magnification to determine the equivalent motion in the object plane. Perfusion of extracellular solution containing 0.2 mM dihydrostreptomycin (DHS; Sigma, Gillingham, UK, and St. Louis, MO) was used to establish what fraction of the resting current originated from the MT channels (Marcotti et al., 2005).

### Temperature Corrections

All experiments on rats and some on gerbils, especially those assaying MT currents, were conducted at room temperature, T = 21–24°C. To determine the effect of temperature on current amplitude, a set of gerbil experiments was performed at both room and body temperature (36°C). The temperature was controlled by a substage heating device with feedback from a thermocouple in the bath and was monitored at the preparation with another digital thermometer. The temperature dependence of the current is described by the temperature coefficient (Q_10_) calculated from:

(2) $${\text{Q}}_{10}={\left({\text{I}}_{2}/{\text{I}}_{1}\right)}^{\left(10/\left(\text{T}2-\text{T}1\right)\right)},$$

where I_1_ and I_2_ are the current amplitudes measured at the lower (T1) and higher (T2) temperatures respectively. Measurements on apical OHCs gave maximum MT currents in 1.3 mM Ca^2+^ of 650 ± 23 pA (n = 5; T1 = 23.2°C) and 1040 ± 41 pA (n = 6; T2 = 35.9°C), from which a Q_10_ of 1.45 was inferred. Similar measurements on the voltage-dependent K^+^ current gave maximum currents of 6.85 ± 0.70 nA (n = 3; T1 = 23.8°C) and 13.56 ± 0.46 nA (n = 5; T2 = 35.8°C) giving a Q_10_ of 1.76. Q_10_ values for the two currents were used to correct all conductances to 36°C.

## Supplemental Information

Document S1. One Figure
