# Supplementary material for: Prestin-Driven Cochlear Amplification Is Not Limited by the Outer Hair Cell Membrane Time Constant
Source: Neuron. 2011 Jun 23;70(6):1143–54. doi: 10.1016/j.neuron.2011.04.024 (PMC3143834; doi:10.1016/j.neuron.2011.04.024)
Supplement: Document S1. One Figure [file mmc1.pdf]

Neuron, Volume *70*

Supplemental Information

Prestin-Driven Cochlear Amplification Is Not Limited by the Outer Hair Cell Membrane Time Constant

Stuart L. Johnson, Maryline Beurg, Walter Marcotti, and Robert Fettiplace

Supplementary Figure 1

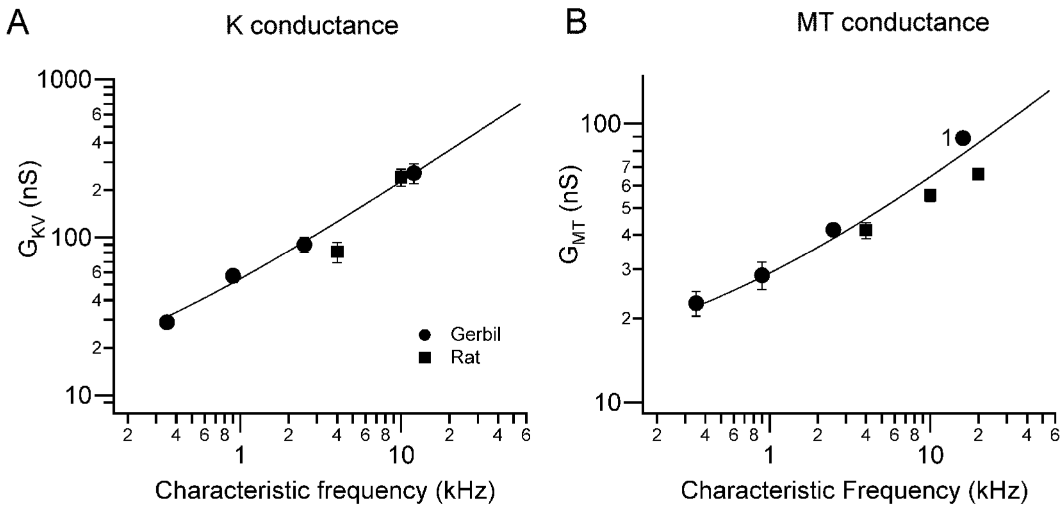

Estimated limit of OHC membrane conductances at high characteristic frequency (CF). (**A**) Peak voltage dependent K^+ conductance (G_{KV}) plotted against CF for gerbil (filled circles) and rat (filled squares); data given in text. The points have been fit with a power function which was extrapolated to 55 kHz, the upper frequency limit in the rat (Müller, 1991), predicting a conductance of 700 nS. (B) Maximum MT conductance (G_{MT}) plotted against CF for gerbil (filled circles) and rat (filled squares); (1) denotes an additional point taken from gerbil data of He et al., (2004) and corrected for low Ca^{2+} endolymph (1.5X) and body temperature (1.6X) using values derived in the text. Extrapolation of the power function fit to 55 kHz gives an upper limit of 130 nS. For the rat, a five-fold apex to base gradient in the total MT conductance for

CFs of 1 – 55 kHz is consistent with increases in the single-channel conductance and number of stereocilia per bundle (Fettiplace, 2009). Both G_{KV} and G_{MT} are given as means ± SEM.

Fettiplace, R. (2009). Defining features of the hair cell mechanoelectrical transducer channel. Pflügers Arch. 458, 1115-23.

He, D.Z., Jia, S., and Dallos, P. (2004). Mechanoelectrical transduction of adult outer hair cells studied in a gerbil hemicochlea. Nature 429, 766-70.

Müller, M. (1991). Frequency representation in the rat cochlea. Hear. Res. 51, 247-254.
